# Network analysis of genes regulated in renal diseases: implications for a molecular-based classification

**DOI:** 10.1186/1471-2105-10-S9-S3

**Published:** 2009-09-17

**Authors:** Suresh K Bhavnani, Felix Eichinger, Sebastian Martini, Paul Saxman, HV Jagadish, Matthias Kretzler

**Affiliations:** 1 Center for Computational Medicine & Bioinformatics, 24 Frank Lloyd Wright Dr., Domino's Farm, Lobby L, Ann Arbor, MI 48109-0738, USA; 2 Michigan Institute for Clinical & Health Research, 24 Frank Lloyd Wright Dr., Domino's Farm, Lobby L, Ann Arbor, MI 48109-0738, USA; 3 Dept. of Internal Medicine, Division of Nephrology, University of Michigan Medical School, 1150 W. Medical Center Drive, MSRB2, SPC 5676 Ann Arbor, MI 48109-5676, USA; 4 Computer Science and Engineering, University of Michigan, 2260 Hayward, Ann Arbor, MI 48109-2121 USA

## Abstract

**Background:**

Chronic renal diseases are currently classified based on morphological similarities such as whether they produce predominantly inflammatory or non-inflammatory responses. However, such classifications do not reliably predict the course of the disease and its response to therapy. In contrast, recent studies in diseases such as breast cancer suggest that a classification which includes molecular information could lead to more accurate diagnoses and prediction of treatment response. This article describes how we extracted gene expression profiles from biopsies of patients with chronic renal diseases, and used network visualizations and associated quantitative measures to rapidly analyze similarities and differences between the diseases.

**Results:**

The analysis revealed three main regularities: (1) Many genes associated with a single disease, and fewer genes associated with many diseases. (2) Unexpected combinations of renal diseases that share relatively large numbers of genes. (3) Uniform concordance in the regulation of all genes in the network.

**Conclusion:**

The overall results suggest the need to define a molecular-based classification of renal diseases, in addition to hypotheses for the unexpected patterns of shared genes and the uniformity in gene concordance. Furthermore, the results demonstrate the utility of network analyses to rapidly understand complex relationships between diseases and regulated genes.

## Background

The rapid development of molecular biology and powerful analytical methods such as network analysis are enabling a shift in our understanding of diseases from a morphological (based on clinical and histological findings) to a molecular basis. This shift in focus has led to improvements in the classification of diseases [1,2]. For example, gene expression analyses have been shown to improve prediction of treatment response in diseases such as breast cancer [3-5] and leukemia [6].

Unfortunately, relatively little is known about how renal diseases are similar and different at the molecular level. Currently, renal diseases are classified largely on morphological similarities. For example, systemic lupus erythematosus (SLE) is classified as a "predominant" *inflammatory *disease based on clinical findings, whereas focal and segmental glomerulosclerosis (FSGS) is classified as a "predominant" *non-inflammatory *disease based on histology. Several studies suggest that such morphology-based classifications could be significantly improved through the analysis of similarities and differences in gene expression, leading to more accurate diagnosis and targeted treatment options [4,6,7].

The analysis of gene expressions in chronic renal disease has either been studied at the level of a single renal disease [8], or by studying gene expressions across all known Mendelian disorders in the OMIM database [9]. The former obviously cannot reveal gene expressions that are common across renal diseases; the latter analyzed renal genes based on limited data, and at a high level (*glomerular *versus *tubular*), and therefore excluded important disease subcategories such as SLE. This article attempts to directly address the lack of understanding about gene expressions in chronic renal diseases. By using new data at the appropriate level of granularity, our goal was to evaluate the current classification of renal diseases, and generate hypotheses about the molecular mechanisms underlying those diseases.

We begin by describing how we assembled a dataset of renal diseases and implicated genes, why and how we represented it using networks, and how we analyzed the networks using visualizations and quantitative measures. We then discuss how the network analysis rapidly revealed unexpected overlaps of genes across the diseases. We conclude by discussing the utility of the network analysis approach to rapidly understand complex relationships, and the need to define a molecular-based classification of chronic renal diseases.

## Methods

Our research began with the question: *What are the molecular similarities and differences between chronic renal diseases? *If gene expressions occur in patterns that match the current classification of renal disease, then we can infer that the current classification is sufficient. However, if diseases have unexpected gene expression similarities, then we can infer that the current classification of renal diseases needs re-evaluation. To address our research question, we made critical decisions regarding *data selection*, *data representation *and *data analysis *as discussed below:

### Data selection

Gene expression data were obtained from 106 patients with one of seven chronic renal diseases (classified in three categories as shown in Table 1) and compared to similar data obtained from biopsies of healthy kidney donors (control). Due to the rarity of three diseases (MCD, TMD, and DN) they currently have very small sample sizes (less than five) in the experimental and/or control conditions.

**Table 1:** Current classification for chronic renal diseases, and the number of patients in the experimental and control groups

**Classification for chronic renal diseases**	**Exp**.	**Ctrl**.
*"Predominant" Inflammatory*		
IgA Nephropathy (**IgAN**)	25	6
Systemic lupus erythematosus (**SLE**)	32	15

*"Predominant" Non-Inflammatory*		
Focal & segmental glomerulosclerosis (**FSGS**)	10	15
Membranous glomerulonephritis (**MGN**)	18	15
Minimal change disease (**MCD**)	4	3
Thin membrane disease (**TMD**)	6	3

*"Predominant" Metabolic*		
Diabetic nephropathy (**DN**)	11	3

**Total**	**106**	**60**

The dataset consisted of biopsies from 106 patients diagnosed with one of 7 renal diseases, and compared with biopsies from 60 healthy patients within the same expression analysis batch. A test of significance between the two groups resulted in 747 genes implicated across the 7 chronic renal diseases.

Microdissected renal tubuli in the biopsies underwent gene expression analysis of 12029 genes in each sample using Affymetrix HG-U133A microarrays. This analysis was done to identify the significantly regulated genes compared to pre-transplant living donor kidney tissues (controls) in each disease. A gene was considered to be significantly regulated in a disease if: (1) the difference of the normalized expression values between control and disease samples was significant at the 0.05 level (after correcting for multiple testing with the false discovery method), and (2) the regulation effect size (as defined by the log2 fold change standardized by a pooled standard deviation) of that gene exceeded +0.3 (for up-regulated) or -0.3 (for down-regulated) when compared to controls. These statistical comparisons between experimental and controls were made within the same expression analysis batch to control for variations in equipment and context. The rigorous controls and tests resulted in a dataset of 747 genes significantly implicated in 7 renal diseases.

### Data representation

Networks [10] have been used to represent a wide range of molecular phenomena related to human diseases [11]. These include networks to represent gene regulation [12], protein-protein interaction (PPI) [13], diseases-gene associations [9,14], and disease-protein associations [15,16]. Idekar and Sharan [15] identified four possible goals for the analysis of such disease networks: (a) identify network properties of disease genes such as the degree of differentially expressed proteins within a PPI network [17], (b) predict the role of disease-causing genes based on their relationship with existing known genes and proteins [18], (c) identify additional genes associated with particular diseases by analyzing the PPI network of known disease-causing proteins [19], and (d) identify highly predictive biomarkers that can be used to classify patients (e.g., those that have or do not have a disease) by identifying sets of biomarkers that are grouped within PPI networks [2]. However, none of these studies have used networks to analyze the similarities and differences between renal diseases based on significantly differentially regulated genes.

**Figure 1:**
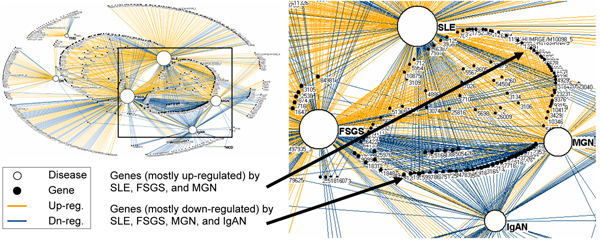
**Bipartite network of genes and renal diseases**. A bipartite network on the left (automatically generated by the *Fruchterman-Rheingold *algorithm [20]) showing the relationship between 7 renal diseases (white nodes), and 747 genes (black nodes). The size of the nodes is proportional to the edges that connect to them. Therefore diseases with many genes have large nodes, whereas diseases with few genes have smaller nodes. The light edges (orange colored) represent up-regulated genes, the dark edges (blue colored) represent down-regulated genes, and gene node labels are the numerical identifiers for each gene from the *Entrez Gene *database. The inset shows that the genes regulated by all four of the high degree diseases (SLE, FSGS, MGN, and IgAN) are mostly down-regulated (shown as having mostly dark edges), whereas those regulated by a subset of the diseases shown (SLE, MGN, and FSGS) are mostly up-regulated (shown as having mostly light edges).

A network is a graph consisting of nodes and edges; nodes represent one or more types of entities (e.g., diseases or genes), and edges between the nodes represent a specific relationship between the entities (e.g., a disease is significantly correlated with a gene). Figure 1 shows a bipartite network (where edges exist only between two different types of entities) of diseases and their implicated genes. As shown, the bipartite network visually represents the explicit relationships between the 7 renal diseases and the 747 expressed genes. Furthermore, the size of a node is proportional to its *degree *(number of edges that connect to that node). Therefore, the larger a node, the more edges it shares with other nodes. Finally, the light edges (orange colored) and dark edges (blue colored) between diseases and genes are significantly up and down regulated respectively.

Networks have three advantages for analyzing complex relationships. (1) They do not require *a priori *assumptions about the data, such as whether the data are hierarchically clustered or contain fuzzy clusters. Instead, by using a simple pair-wise representation of nodes and edges, networks enable rapid discovery of complex relationships using a single representation. (2) The specificity provided by the pair-wise representation between nodes can reveal details of relationships, for example how specific diseases are connected to specific genes. (3) They can be rapidly visualized and analyzed using a set of network algorithms to reveal global regularities in the data. For example, Figure 1 shows how a *force-directed *layout algorithm [20] helps to visualize the relationship between diseases and genes. The algorithm pulls together nodes that are tightly connected, and pushes apart nodes that are not. As shown, the result is that diseases that share genes (e.g., FSGS and SLE in the center of Figure 1) are placed close to each other, and close to their implicated genes. Given these advantages, we used networks to explore the relationship between renal diseases and their implicated genes.

### Data analysis

The insights from the network visualizations were further analyzed using two standard network analysis methods. (1) To quantitatively analyze the overall network topology observed in the visualization, we calculated the *degree *of each gene (number of diseases implicated with a gene) and plotted the *degree distribution *of the genes. (2) To understand more clearly how diseases had common gene expressions, we transformed the bipartite network to inspect only how the diseases shared genes in a method called a *one-mode projection *[10]. Here, all gene nodes were removed, and a weighted edge was placed between two diseases if they shared one or more genes. The resulting network visually represented how pairs of diseases shared gene expressions, revealing how two diseases share more or less genes than expected in comparison to the current classification of renal diseases. The networks were created using *Pajek *(version 1.23) [21].

## Results

The bipartite network visualization revealed three critical patterns related to renal diseases and genes:

### Many specific genes, fewer non-specific genes

As shown in Figure 1, there are a large number of *specific *genes in the periphery of the network that are connected to a single disease. These low degree nodes have been pushed out to the periphery by the force-directed layout algorithm due to their low connectivity with many diseases. In contrast, there are relatively fewer *non-specific *genes that are in the center of the network due to their high connectivity to many diseases.

**Figure 2:**
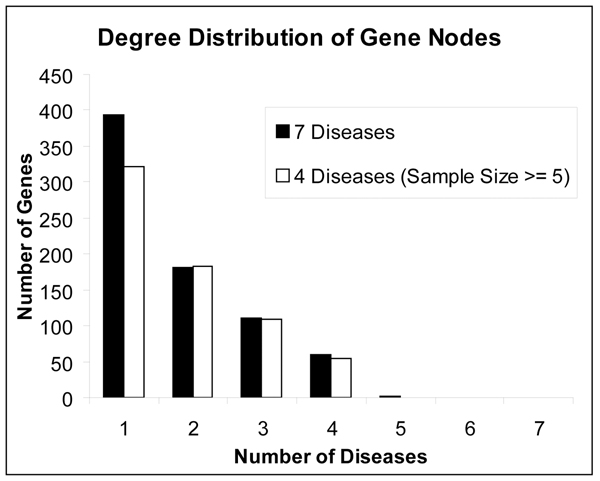
**Degree distribution of gene nodes**. Degree distribution of the gene nodes for 7 diseases in the network showing more than half of the genes implicated in one disease (specific genes), two genes implicated in 5 diseases (non-specific genes), and none of the genes implicated in all diseases. The overall pattern of many specific genes and fewer non-specific genes holds even when 3 diseases with small sample sizes were excluded from the dataset.

The degree distribution of genes (Figure 2) provides a quantitative basis for this observation: more than half (54%) of the total 747 genes are implicated in just one disease, only two genes (0.3%) are implicated in 5 diseases, and none of the genes are implicated in 6 or all of the 7 diseases. This result provided the first glimpse into the pattern of overlap between diseases and implicated genes. The network in Figure 1 also shows the low number of overall genes for MCD, TMD, and DN which have been pushed to the periphery of the graph due to their low gene overlap. Because each of these diseases has a low sample size (as shown in Table 1) we removed them from the dataset to test its effect on the distribution. As shown in Figure 2, while the number of specific genes drops, the distribution still shows an overall pattern of many specific genes and fewer non-specific genes.

### Relationship between diseases, genes, and regulation type

The network visualization revealed a multidimensional relationship between diseases, genes, and regulation type. As shown in Figure 1, there exist three disease sets which share a disproportionately large number of genes: (a) the four dominant diseases (SLE, FSGS, MGN, IgAN) on the right hand side of the network share 52 (88%) of the total 4-degree genes. These genes are mainly down-regulated. (b) A proper subset of the above disease set (SLE, FSGS, MGN) share 88 (79%) of the total 3-degree genes. These genes are mainly up-regulated. (c) A pair of diseases (SLE, FSGS) which overlap with the above sets share 130 (72%) of the total 2-degree genes. These genes are mainly up-regulated.

**Figure 3:**
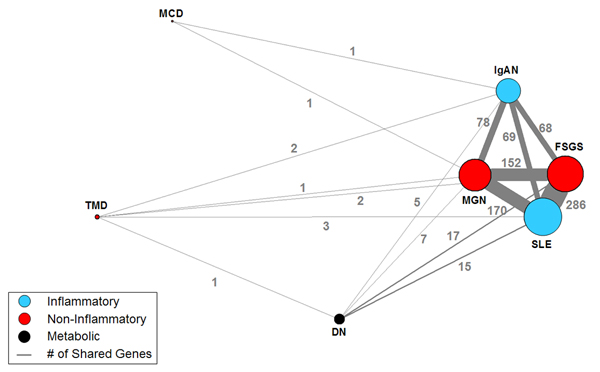
**One-mode projection on renal diseases**. A one-mode projection on diseases of the network shown in Figure 1, showing how pairs of diseases share genes. The edge thickness is proportional to the number of shared genes (shown as numbers on the edges), and the size of the nodes is proportional to the sum of the edge weights incident to that node. Removal of diseases with small sample sizes (TMD, DN, and MCD) has no effect on the relationship between the other diseases.

The gene expression overlap between diseases is shown more clearly by a one-mode projection on diseases. As shown in Figure 3, the one-mode projection removed the gene nodes, and placed edges between the diseases to correspond with how many genes each pair shares. The one-mode projection shows three dominant pairs of diseases (SLE-FGS, SLE-MGN, FGS-MGN) that share many genes. While the projection is not designed to reveal whether more than two diseases share the *same *genes, the network clearly shows the dominant relationship between SLE, FGS, MGN, and a less dominant relationship with IgAN. Future studies with larger samples of TMD, DN, and MCD should reveal how they relate to the other renal diseases.

### Uniform concordance in gene regulation

All the genes in the network, regardless of degree, are concordantly regulated. In other words, no gene was up-regulated in one disease, and down-regulated in another. This uniform concordance in gene regulation can be seen by the large areas of uniform color for edges connecting to high degree genes. Given the 100% uniformity of gene concordance, we re-examined the data to check for programmatic and bias errors, but found none. Furthermore, we examined another dataset containing biopsies from patients with acute renal failure. When the two data sets were merged, we found two genes that were discordantly regulated. This suggests that the uniformity in gene regulation within chronic renal diseases (presented here) is most probably the result of similarity in biological mechanisms across chronic renal diseases, rather than a selection bias or error. Future research should further verify this conclusion.

## Discussion

Given that the data consisted only of the tubular compartment of renal biopsies, we expected to find a large number of non-specific (shared) genes. Instead, we found relatively few non-specific renal genes associated with a large number of diseases. This relationship held even when we removed diseases with a low sample size. It is important to note that the many specific genes in our dataset could be implicated in other renal diseases not included in our study, and therefore could be non-specific with respect to a wider scope of diseases. Network analysis therefore helped to identify which genes might be involved in molecular mechanisms that are specific to a disease, and which genes are involved in common pathways activated in combinations of chronic renal diseases. This approach could guide future research to identify specific and general drug therapies to treat kidney diseases.

Besides the distribution of specific and non-specific genes, the network analysis also revealed patterns of molecular similarity between diseases which do not match the current morphology-based classification of renal diseases. As shown in the first column of Table 1, SLE and IgAN belong to the class of *inflammatory *diseases. However, the network analysis revealed that SLE shares many more genes with FSGS and MGN (from the *non-inflammatory *class), compared to IgAN (from its own class). While molecular similarities between non-inflammatory and inflammatory renal diseases have been previously reported [22,23] the unexpected finding was the strength of the association with members outside its class. Similarly, IgAN shares an equal number of genes with SLE (from its own class) as it does with FSGS, and MGN (from the *non-inflammatory *class). These results suggest that the current morphology-based classification of renal diseases does not match the pattern of shared tubulo-interstitial gene expression, and therefore motivates future research to define a new molecular-based classification of renal diseases.

The above result also motivates future research to investigate how genes common to sets of disease can guide the identification of existing or new gene regulatory pathways [16]. For example, in a preliminary analysis we used *canonical pathways *(developed by Ingenuity^® ^Systems) to search for existing regulatory pathways that best matched the genes shared by the three dominant disease sets. For the 59 genes shared by the disease set FSGS, MGN and IgAN, the search retrieved 45 canonical pathways that were significant at the 0.01 level. Many of these pathways (e.g., TGF-β, JAK/STAT, NF-κB and VEGF) were experimentally-verified in these diseases. For example, involvement of the VEGF pathway is suggestive of vascular rarefication as an underlying driving force for ischemic damage in renal failure [24], and is a potential biomarker for progressive renal disease. However, it is possible that shared genes in our network do not match known pathways, which would suggest the existence of new pathways yet to be discovered. These new pathways could be important in understanding the pathophysiology of the diseases.

Finally, the concordance in gene regulation and the fact that genes in the three dominant disease sets are either mostly up or down-regulated suggest that shared mechanisms have identical effects on gene regulation. This can be further investigated in the future by analyzing the properties of the shared genes using categories from the Gene Ontology database. Patterns in how the gene categories relate to different disease sets should lead to an understanding of this and other phenomena related to the type of gene regulation. The network analysis therefore led to several testable hypotheses about the underlying pathophysiological mechanisms involved in chronic renal disease.

## Conclusion

While networks have been used to analyze a wide range of molecular phenomena, they have not been used to analyze how genes are implicated in multiple renal diseases. Our analysis has enabled us to question the adequacy of the existing morphological based taxonomy of renal diseases. Furthermore, the analysis rapidly revealed useful biological insights, without requiring additional filtering to reveal complex but understandable relationships. This could be because the network was of medium size and density compared to many large networks, such as the Gnutella peer-to-peer file sharing network [25]. In addition, the resulting network quickly revealed overlapping, nested, and subset groups *from the same *representation, a result that would be difficult using traditional data mining techniques. However, it is important to note that like most data mining techniques, network analysis is essentially an exploratory tool, and most useful for generating hypotheses, which need rigorous testing using other techniques to arrive at definitive answers.

The limitation of the current analysis is the small sample sizes for three diseases, which new data will soon address. However, similar to the *Diseasome *project [9], studies that attempt to analyze gene expressions of many diseases simultaneously often have to deal with incomplete data. Networks are surprisingly useful for incomplete data because they enable us to visually inspect the data, and make appropriate choices for filtering and interpretation.

Our future research includes: (1) Using categories from the Gene Ontology database to annotate the genes in the bipartite network, with the goal of understanding why sets of shared genes have similar regulation type. (2) Analyzing the data using additional network analytical techniques [26], such as random network comparison [27,28] and fuzzy cluster analysis [29,30]. (3) Analyzing a network consisting of individual patients and expressed genes. The goal of analyzing individual patients is to construct a new classification of renal diseases using a bottom-up approach without the use of *a priori *disease classifications as was done in the current study. As previous research on biomarkers in renal diseases have stated, gene expression data should lead to a systematically constructed molecular-based classification, resulting in the identification of more targeted treatments for patients with chronic renal disease.

## Competing interests

The authors declare that they have no competing interests.

## Authors' contributions

SB conceived the initial idea to analyze renal diseases and regulated genes using bipartite and one-mode networks, and constructed them; FE extracted and formatted the data; SB, SM, FE, and PS analyzed the networks; MK supervised the project. SB, FE, SM, PS, HJ, MK wrote, discussed, revised, and approved the final manuscript.

## References

[B1] Golub T, Slonim D, Tamayo P, Huard C, Gaasenbeek M, Mesirov J, Coller H, Loh M, Downing J, Caligiuri M (1999). Molecular Classification of Cancer: Class Discovery and Class Prediction by Gene Expression Monitoring. Science.

[B2] Chuang H, Lee E, Liu Y, Lee D, Ideker T (2007). Network-based classification of breast cancer metastasis. Molecular Systems Biology.

[B3] Wulfkuhle JD, Speer R, Pierobon M, Laird J, Espina V, Deng J, Mammano E, Yang SX, Swain SM, Nitti D (2008). Multiplexed Cell Signaling Analysis of Human Breast Cancer Applications for Personalized Therapy. Journal of Proteome Research.

[B4] van 't Veer LJ, Dai H, Vijver MJ van de, He YD, Hart AA, Mao M, Peterse HL, Kooy K van der, Marton MJ, Witteveen AT (2002). Gene expression profiling predicts clinical outcome of breast cancer. Nature.

[B5] Hall P, Ploner A, Bjöhle J, Huang F, Lin C-Y, Liu E, Miller L, Nordgren H, Pawitan Y, Shaw P (2006). Hormone-replacement therapy influences gene expression profiles and is associated with breast-cancer prognosis: a cohort study. BMC Medicine.

[B6] Cario G, Stanulla M, Fine B, Teuffel O, Neuhoff N, Schrauder A, Flohr T, Schafer B, Bartram C, Welte K (2005). Distinct gene expression profiles determine molecular treatment response in childhood acute lymphoblastic leukemia. Blood.

[B7] Loscalzo J, Kohane I, Barabasi A-L (2007). Human disease classification in the postgenomic era: A complex systems approach to human pathobiology. Mol Syst Biol.

[B8] Martini S, Eichinger F, Nair V, Kretzler M (2008). Defining human diabetic nephropathy on the molecular level: Integration of transcriptomic profiles with biological knowledge. Reviews in Endocrine & Metabolic Disorders.

[B9] Goh K, Cusick M, Valle D, Childs B, Vidal M, Barabási A (2007). The human disease network. Proc Natl Acad Sci U S A.

[B10] Newman M (2003). The structure and function of complex networks. SIAM Review.

[B11] Junker BH, Schreiber F (2008). Analysis of Biological Networks (Wiley Series in Bioinformatics).

[B12] Albert Rk (2004). Boolean Modeling of Genetic Regulatory Networks. Complex Networks.

[B13] Schwikowski B, Uetz P, Fields S (2000). A network of protein-protein interactions in yeast. Nat Biotechnol.

[B14] Oti M, Brunner H (2007). The modular nature of genetic diseases. Clinical genetics.

[B15] Ideker T, Sharan R (2008). Protein networks in disease. Genome Research.

[B16] Sam L, Liu Y, Li J, Friedman C, Lussier Y (2007). Discovery of protein interaction networks shared by diseases. Pacific Symposium on Biocomputing.

[B17] Wachi S, Yoneda K, Wu R (2005). Interactome-transcriptome analysis reveals the high centrality of genes differentially expressed in lung cancer tissues. Bioinformatics.

[B18] Oti M, Snel B, Huynen M, Brunner H (2006). Predicting disease genes using protein-protein interactions. Journal of medical genetics.

[B19] Pujana M, Han J, Starita L, Stevens K, Tewari M, Ahn J, Rennert G, Moreno V, Kirchhoff T, Gold B (2007). Network modeling links breast cancer susceptibility and centrosome dysfunction. Nature genetics.

[B20] Fruchterman T, Reingold E (1991). Graph drawing by force-directed placement. Software: Practice and Experience.

[B21] Batagelj V, Mrvar A (2003). Pajek – analysis and visualization of large networks. Graph Drawing Software.

[B22] Ivanova L, Rudolph P, Shilov Y, Gieseler F, Alm P, Tareeva I, Proppe D (2001). Correlation between the expression of DNA topoisomerases I and IIalpha and clinical parameters in kidney disease. American journal of kidney diseases: the official journal of the National Kidney Foundation.

[B23] Preston G, Waga I, Alcorta D, Sasai H, Munger W, Sullivan P, Phillips B, Jennette J, Falk R (2004). Gene expression profiles of circulating leukocytes correlate with renal disease activity in IgA nephropathy. Kidney international.

[B24] Lindenmeyer M, Kretzler M, Boucherot A, Berra S, Yasuda Y, Henger A, Eichinger F, Gaiser S, Schmid H, Rastaldi M (2007). Interstitial vascular rarefaction and reduced VEGF-A expression in human diabetic nephropathy. Journal of the American Society of Nephrology.

[B25] Ripeanu M, Iamnitchi A, Foster I (2002). Mapping the Gnutella Network. IEEE Internet Computing.

[B26] Costa, Rodrigues FA, Travieso G, Boas V (2007). Characterization of complex networks: A survey of measurements. Advances in Physics.

[B27] Wang X, Chen G (2003). Complex networks: small-world, scale-free and beyond. Circuits and Systems Magazine, IEEE.

[B28] Strogatz SH (2001). Exploring complex networks. Nature.

[B29] Zhang S, Wang R, Zhang X (2007). Identification of overlapping community structure in complex networks using fuzzy cc-means clustering. Physica A: Statistical Mechanics and its Applications.

[B30] Reichardt Jo, Bornholdt S (2004). Detecting Fuzzy Community Structures in Complex Networks with a Potts Model. Physical Review Letters.

